# Exploring Deep Learning to Predict Coconut Milk Adulteration Using FT-NIR and Micro-NIR Spectroscopy

**DOI:** 10.3390/s24072362

**Published:** 2024-04-08

**Authors:** Agustami Sitorus, Ravipat Lapcharoensuk

**Affiliations:** Department of Agricultural Engineering, School of Engineering, King Mongkut’s Institute of Technology Ladkrabang, Bangkok 10520, Thailand

**Keywords:** adulteration, chemometric, coconut milk, deep learning, food, non-destructive

## Abstract

Accurately identifying adulterants in agriculture and food products is associated with preventing food safety and commercial fraud activities. However, a rapid, accurate, and robust prediction model for adulteration detection is hard to achieve in practice. Therefore, this study aimed to explore deep-learning algorithms as an approach to accurately identify the level of adulterated coconut milk using two types of NIR spectrophotometer, including benchtop FT-NIR and portable Micro-NIR. Coconut milk adulteration samples came from deliberate adulteration with corn flour and tapioca starch in the 1 to 50% range. A total of four types of deep-learning algorithm architecture that were self-modified to a one-dimensional framework were developed and tested to the NIR dataset, including simple CNN, S-AlexNET, ResNET, and GoogleNET. The results confirmed the feasibility of deep-learning algorithms for predicting the degree of coconut milk adulteration by corn flour and tapioca starch using NIR spectra with reliable performance (*R*^2^ of 0.886–0.999, RMSE of 0.370–6.108%, and Bias of −0.176–1.481). Furthermore, the ratio of percent deviation (RPD) of all algorithms with all types of NIR spectrophotometers indicates an excellent capability for quantitative predictions for any application (RPD > 8.1) except for case predicting tapioca starch, using FT-NIR by ResNET (RPD < 3.0). This study demonstrated the feasibility of using deep-learning algorithms and NIR spectral data as a rapid, accurate, robust, and non-destructive way to evaluate coconut milk adulterants. Last but not least, Micro-NIR is more promising than FT-NIR in predicting coconut milk adulteration from solid adulterants, and it is portable for in situ measurements in the future.

## 1. Introduction

Adulteration in agriculture and food products is an essential safety and control area requiring rapid, accurate, robust, and automated methods for detecting, identifying, and quantifying adulteration, including coconut milk products. Coconut milk is generally extracted from grated coconut meat after pressing or squeezing with or without the addition of water. Coconut milk has been used as a major ingredient in several cuisines, such as curries and desserts [1]. There are two common reasons for adulteration in coconut milk products. The first reason is to increase production volume and reduce costs by adding tap water or old coconut water to coconut milk. The second reason is an attempt to boost the apparent carbohydrate content by adding corn flour.

Accurately identifying adulterants is important for controlling coconut milk product adulteration. The main content of coconut milk (moisture, total fat, carbohydrates, protein, and ash) will be changed when mixed with other materials. As reported by Lakshanasomya et al. [2], laboratory testing can measure the total solids and total fat in coconut milk by drying it in a hot air oven or using a vacuum oven, which takes more than 2 h to prepare one sample. Although accurate, this method is time-consuming and requires complicated sample pre-treatments and well-trained technicians, so it cannot be relied on to carry out rapid monitoring. Near-infrared (NIR) and mid-infrared spectroscopy have gained considerable interest among the approaches to physical properties, particularly for detecting adulteration in many agricultural and food products. Compared with the above methods, NIR and mid-infrared spectroscopy are analytical techniques with the advantages of rapid response in real time, simplicity in testing, and are non-destructive. However, this method requires the development of a calibration model before it can be used to make predictions.

Several efforts to develop calibration models have been created using a chemometrics approach to achieve better performance prediction of coconut milk adulteration based on NIR spectroscopy. For instance, Azlin-Hashim et al. [3] employed partial least squares (PLS) regression to quantitatively determine the concentration of corn flour in the coconut milk using an FT-IR spectrometer. This study used spectroscopic techniques in mid-infrared zones combined with classical chemometrics. Although advantageous, classical chemometric analysis is frequently criticized for its requirement of expertise and subjectivity in elaborating spectral data, including selecting an excellent preprocessing method based on what worked well on a previous data set and how to highlight important spectral regions [4,5]. Therefore, Sitorus and Lapcharoensuk [6] adopted a machine-learning algorithm with automatic preprocessing to predict water in coconut milk using an FT-NIR spectrometer. Al-Awadhi and Deshmukh [7] utilized linear discriminant analysis (LDA) and K-nearest neighbors (KNN) from machine learning as a classifier to detect water in coconut milk using an FTIR spectrometer. They succeeded in improving model accuracy but were observed to be complicated structures that were difficult to train and with apparent risks of over-fitting. Moreover, although robust and accurate, these strategies have drawbacks related to data dimensionality and higher entropy apart from efforts and practical feasibility [5,8,9]. Furthermore, efforts related to learning representations of the data that identify and highlight the underlying explanatory factors hidden in the data are still challenging in machine-learning applications [10]. Consequently, some studies are probing for a shift in the paradigm toward applying deep learning to resolve the issues related to classical and feed-forward neural network approaches.

Deep learning is a branch of machine learning that begins with images as input and learns to identify patterns within their spatial dimensions. Deep learning consists of multiple processing layers to automatically learn complex representations from data without introducing hand-coded rules or human domain knowledge. Among deep-learning algorithms, convolutional neural networks (CNNs) are presently one of the most trending models since they do not require manual feature extraction and have several network architecture types. CNNs are constructed with a series of convolutional layers that act as feature extractors, followed by fully connected layers at the end of the network that serve as predictors. For processing NIR spectral data, it was also recently seen to be useful for one-dimensional (1D) spectroscopy data, as well as for regression tasks wherein this supervised approach could perform both feature extraction and learning related to features of interest [8]. Presently, CNN techniques are developing rapidly so many network architecture variants are found for various analysis purposes, such as AlexNet, ResNET, GoogLeNet, etc. [11]. Furthermore, in the case of chemometrics data, the use of CNN can enable training on smaller weights, thereby lowering data complexity as opposed to fully connected or feed-forward neural networks. Some of the advantages of CNN are a reduction in neuron interdependence, adaptability to datasets beyond the training, and reduced risk of over-fitting, which is a common criticism in feed-forward networks. Moreover, some researchers [4,8] note that CNN can eventually simplify preprocessing and model development, thereby reducing the complexity of model development and improving accurate and robust model predictions. Recent papers on the utilization of CNN for NIR spectroscopy, especially in agricultural and food products, have reported adulteration in coffee products (Nallan Chakravartula et al. [4]), adulteration in infant formula products (Liu et al. [12]), adulteration in dairy products (Said et al. [13]), and adulteration in minced beef products (Weng et al. [14]). This shows the increasing number of studies using the CNN algorithm in NIR-based adulteration detection for agricultural and food products.

To the best of our knowledge, even though coconut milk adulteration was investigated with another adulterant material and another type of spectroscopy [3,6], no study explored deep learning as advanced computational algorithms for quantifying adulterants by two types of NIR spectroscopy, including benchtop FT-NIR and portable Micro-NIR and by two types of solid adulterants, including corn flour and tapioca starch. Therefore, the objective of this study was to bridge the gap between advanced perceptual sensors from NIR spectroscopy and data science by developing and testing the performance of four types of regressor architecture CNN of deep-learning to detect coconut milk adulteration from corn flour and tapioca starch.

## 2. Materials and Methods

### 2.1. Sample Collection

This study comprised two parts. The first part was to detect the level of coconut milk adulteration by corn flour and tapioca starch using a benchtop FT-NIR spectrometer. The second part was to detect coconut milk adulteration levels by corn flour and tapioca starch utilizing a portable Micro-NIR spectrometer. For this purpose, the spectra of two potential adulterants (corn flour and tapioca starch) and coconut milk were collected. The adulterants were purchased from a grocery market around Lat Krabang, Thailand. Adulterant samples were stored at room temperature under clean and dry conditions. Coconut milk is the liquid extracted from mature coconut fruit’s endosperm, with no added diluents like water or other materials. Coconut milk was obtained from traditional markets (Lad Krabang, Thailand) on 6 January 2024 and processed on the same day. 

The coconut milk samples were adulterated by mixing the identified adulterant in the range of 1–50% (*w*/*w*) at different adulteration levels (approximately 1%, 2%, 3%, 4%, 5%, 6%, 7%, 8%, 9%, 10%, 15%, 20%, 30%, 40%, and 50%). The reasons for selecting adulteration levels in this study were as follows. Selecting different mixing levels can help represent various levels of mixing severity. Covering low mixing levels (1%) to high levels (50%) can provide a more complete view of the range of possible mixing situations that will be encountered in practice. Relevance to practical applications where mixing levels with minimum conditions for agriculture and food products are more likely to occur frequently in practical situations in the field so that they have a more significant impact on this study. Also, selecting various mixing levels can help test the model’s calibration ability to detect mixing at various severity levels. This can help identify the extent to which the model is reliable in identifying mixing at different levels.

After the coconut milk samples had been intentionally adulterated to the level of conditions specified above, all samples were subsequently preserved in glass bottles at room temperature after being mixed in a glass beaker at a speed of 200 rpm for 1 min and allowed to equilibrate to ±25 °C before scanning. Ten samples were prepared at each level of adulteration and each of adulterant. Therefore, a total of 150 samples were prepared per type of adulterant. The total data analyzed in this study were higher than in the suggestion by Manley [15] that developing regression models, which must have at least more than 100 spectra to obtain a reliable model, has exceeded that.

### 2.2. NIR Spectroscopy Data Acquisition

**Figure 1:**
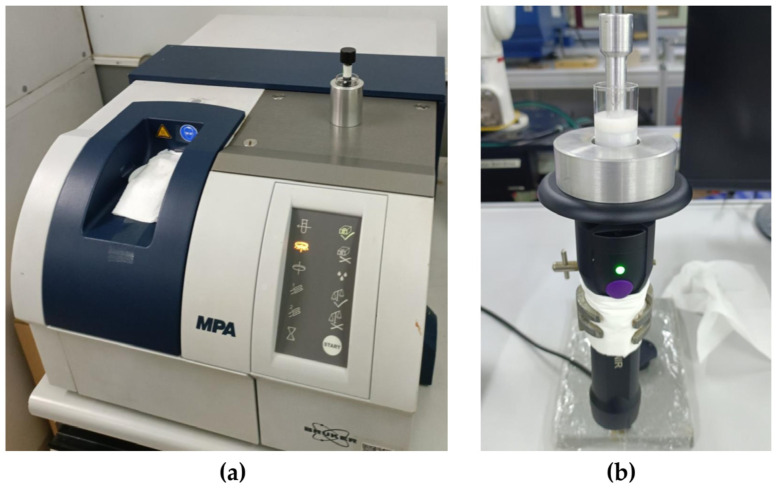
Detection conditions for scanning NIR data by (**a**) FT-NIR and (**b**) Micro-NIR.

NIR spectra were measured using a benchtop FT-NIR spectrometer (Bruker Ltd., Ettlingen, Germany) and a portable Micro-NIR spectrometer (MicroNIR OnSite-W, VIAVI Solutions Inc., Chandler, AZ, USA). Spectra in the 12,500–4000 cm^−1^ (800–2500 nm) region were recorded using the benchtop FT-NIR (Figure 1a) with an average spectrum of 32 scans into one spectrum at a resolution of 8 cm^−1^. Secondly, portable Micro-NIR (Figure 1b) scanned in the spectra range from 908 to 1676 nm (11,013–5967 cm^−1^). The spectral resolution was set to 6.2 nm. The integration time and scan count were 10 ms and 100, respectively. FT-NIR and Micro-NIR were used together to obtain more complete and comprehensive information about NIR spectra of coconut milk samples adulterated with two potential adulterants (corn flour and tapioca starch).

A sample of coconut milk (1 mL) that was intentionally adulterated was taken to test a glass vial with a diameter of 20 mm and a height of 43 mm. After that, an aluminum reflector (with a path length of 0.35 mm) was also put in a test glass vial and placed on top of the benchtop FT-NIR and portable Micro-NIR spectrometer. Scanning was performed triplicated at the same spatial locations for each sample, accumulating 3 scanning ×10 samples ×15 level adulteration. All of the measurements were taken at room temperature (±25 °C). Scanning was performed in absorption mode (log 1/R).

### 2.3. Data Handling for Modelling

The whole dataset in this study is 1800 NIR spectra consisting of 900 spectra scanned by benchtop FT-NIR and 900 spectra scanned by portable Micro-NIR. Each NIR spectrum acquired from these two instruments consists of 450 spectra from adulteration coconut milk by corn flour and 450 spectra from adulteration coconut milk by tapioca starch. From the NIR spectra data, each adulteration was split into training and testing subsets. The training data were used to develop a deep-learning model. Then, the models were applied to the test dataset to assess the predictive abilities of the models for predicting the adulteration level of coconut milk by corn flour and tapioca starch. Table 1 summarizes adulteration coconut milk data spectra, including NIR spectra data collected from benchtop FT-NIR and portable Micro-NIR. The number of training (70%) and testing (30%) data used in this study was 315:135 (separated using the random splitting method with a random state of 42).

### 2.4. Deep-Learning Model Development

**Figure 2:**
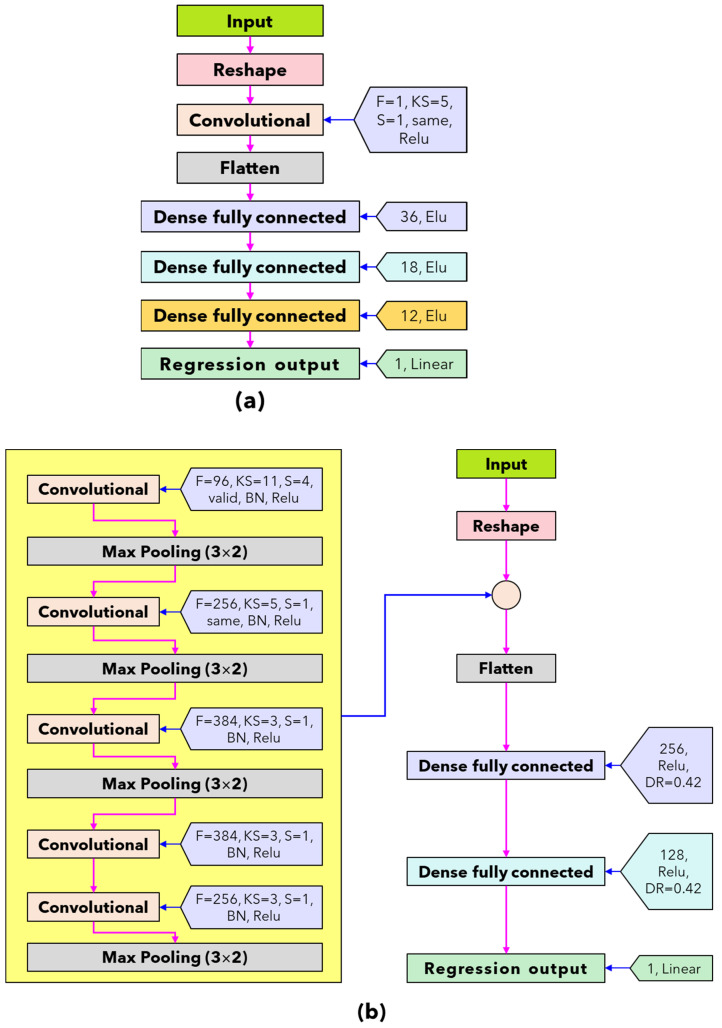

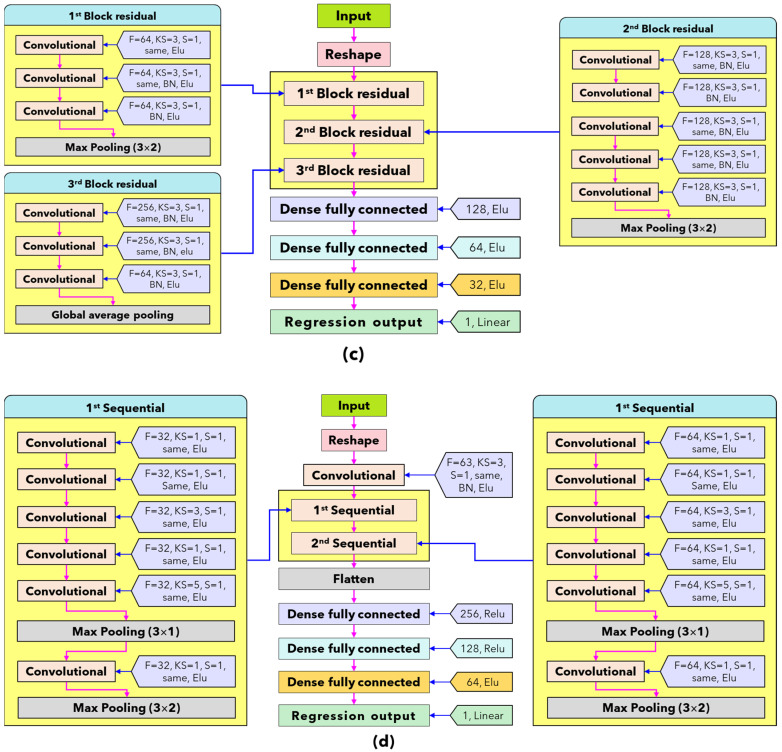
Architecture and parameters of the proposed algorithms in this study. (**a**) Simple CNN regressor; (**b**) S-AlexNet regressor; (**c**) ResNET regressor; and (**d**) GoogleNET regressor.

For analyzing the NIR datasets with deep-learning models, a unique structure is required to provide suitable training and improve the feature extraction process. The proposed deep-learning regressor model for predicting coconut milk adulteration in this study uses four types of network architecture that are modified to a one-dimensional framework was developed, including simple convolutional neural network (Simple CNN), A-AlexNet, ResNET, and GoogleNET, which are presented in Figure 2. The reason for using simple CNN was based on its effectiveness and efficiency in processing data, which is particularly suitable for relatively simple datasets [5]. S-AlexNet was selected as an adaptation of the successful AlexNet architecture, offering a lighter architecture suitable for smaller datasets [16]. ResNet was chosen for its ability to address the vanishing gradient problem and enable the training of deeper models, making it suitable for complex datasets requiring deep feature representations [17]. GoogleNet was selected for its innovative architecture, particularly the efficient use of inception modules for feature extraction, making it ideal for complex datasets requiring multi-scale feature representations [18]. In addition, the original spectral data were preprocessed using standard normal variate (SNV) to obtain input features spectrum to have zero mean and standard deviation of one. The SNV preprocessing effectively reduced specific noise appearing in the spectral data due to the effect of ambient light, the spectrometer used, and the type of lamp [19].

All types of network architecture model deep-learning training procedures in this study were performed using Adam optimizer. In the simple CNN network architecture (Figure 2a), the number of batches, epochs per running, validation split, and learning rate are 128, 1000, 10%, and 5 × 10^−3^, respectively. In the S-AlexNET network architecture (Figure 2b), the number of batches, epoch per running, validation split, and learning rate are 16, 300, 10%, and 10^−5^, respectively. In the ResNET network architecture (Figure 2c), the number of batches, epochs per running, validation split, and learning rate are 160, 1000, 10%, and 10^−5^, respectively. In the GoogleNET network architecture (Figure 2d), the number of batches, epochs per running, validation split, and learning rate are 160, 1000, 10%, and 10^−5^, respectively. The total running time is 11 times with early stopping patience of 200 epochs for simple CNN, ResNET, and GoogleNET and 300 epochs for S-AlexNET. All network architectures use random split validation at the training stage with 10% from the training dataset (10% × 315 = 32) with a random state 42. After that, the best deep-learning regression was evaluated with a testing dataset of as much as 135.

In this study, all of the deep-learning algorithms were programmed on the JupyterLab interface using the open source platform Python version 6.5.4, Keras library version 2.13.1 [20] with TensorFlow version 2.13.0 backend [21]. The CPU is Intel (R) core (TM) i9-13900H CPU @ 2.60 Ghz, and the graphics card is NVIDIA Geforce RTX 4060.

### 2.5. Performance Model Evaluation

The performance of models was assessed by the coefficient of determination, root-mean-square error, Bias, and the ratio of percent deviation (RPD), which were calculated by Equations (1)–(4). The higher coefficient of determination values and lower root-mean-square error and Bias indicate an accurate model. In food adulteration, spectral modeling follows predicting grain chemical composition content; RPD  <  3.0 shows a poor and unreliable model; 3.1  <  RPD  <  4.9 indicates a fair model (just for screening); 5.0  <  RPD  <  6.4 shows a good model for quality control; 6.5  <  RPD  <  8.0 indicates very good model for process control, and RPD  >  8.1 indicates an excellent capability of the model for quantitative predictions for any application [22].

(1) $$R^{2}=1-\frac{\sum_{i=1}^{n}{\left(E_{i}-P_{i}\right)}^{2}}{\sum_{i=1}^{n}{\left(E_{i}-\bar{E}\right)}^{2}}$$

(2) $$\mathrm{RMSE}=\sqrt{\frac{\sum_{i=1}^{n}{\left(E_{i}-P_{i}\right)}^{2}}{N}}$$

(3) $$\mathrm{Bias}=\frac{\sum_{i=1}^{n}\left(E_{i}-P_{i}\right)}{N}$$

(4) $$\mathrm{RPD}=\frac{1}{\sqrt{1-R^{2}}}$$

where *R*^2^ is the coefficient of determination; RMSE is root-mean-square error; RPD is a ratio of percent deviation; *E_i_* is the existing value for point to-*i*; *P_i_* is the prediction value for point to-*i*; *N* is the number of samples, and $\bar{E}$ is average of existing value. 

To interpret the obtained models, this study proposes a method to visualize the regression coefficients of neural networks numerically. This method is modified from Cui and Fearn [8] and can be used for linear predictors. As a black box that maps the input spectrum, the predictor was treated to a single prediction value using Equation (5). The single total weight is calculated using Equation (6) (finite difference approximation) from the main Equation (7).

(5) $$w=\frac{\sum_{i=1}^{N}w_{i}}{N}$$

(6) $$w_{i}=\frac{f\left(x_{1}+\varepsilon ,\ldots x_{i}+\varepsilon ,\ldots ,x_{n}+\varepsilon \right)-f(x_{1},\ldots x_{i},\ldots ,x_{n})}{\varepsilon }$$

(7) $$f\left(x\right)=\left[\sum_{i=1}^{N}\left(w_{i}x_{i}\right)\right]+b$$

where *w* is the average of weight; *w_i_* is weight to-*i*; *N* is the number of samples; *x*_1_ to *x_n_* is the absorbance of feature NIR spectra; *b* is the intercept, and *ε* is perturbation coefficient (10^−6^).

## 3. Results

### 3.1. NIR Spectra Features

**Figure 3:**
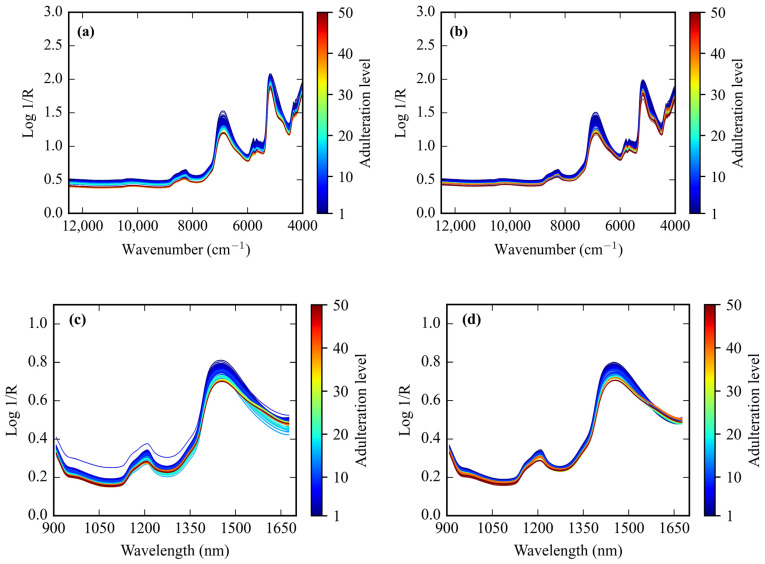
The original NIR spectroscopy data. Spectra by benchtop FT-NIR from coconut milk adulteration by (**a**) corn flour and (**b**) tapioca starch. Spectra by portable Micro-NIR for coconut milk adulteration by (**c**) corn flour and (**d**) tapioca starch.

NIR spectra to detect coconut milk adulteration in this study were acquired using two spectrophotometers, benchtop FT-NIR (450 spectra) and portable Micro-NIR (450 spectra). The original spectrum from the benchtop FT-NIR is presented in Figure 3a,b, and the original spectrum from the portable Micro-NIR is shown in Figure 3c,d. Figure 3a,c shows the FT-NIR and Micro-NIR spectra of coconut milk adulteration by corn flour from 1–50%. Meanwhile, the spectrum of FT-NIR and Micro-NIR of coconut milk adulteration by tapioca starch from 1–50% is shown in Figure 3b,d. The NIR spectrum indicates the presence of organic materials resulting from the interaction of molecular bonds of XH with the incident radiation from coconut milk, corn flour, and tapioca starch. The absorption peak positions appear almost indistinguishable, with only slight differences in absorption between adulteration levels. These bonds are subject to vibrational energy changes, including stretching and bending. The presence of strong water absorbance bands from FT-NIR was observed at around 5176 cm^−1^ (1932 nm) and 6889 cm^−1^ (1452 nm) because of the OH combination and its first overtone for both adulterant corn flour and tapioca starch. When utilizing Micro-NIR, strong water absorbance bands were identified around 1447 nm (6911 cm^−1^) as an OH first overtone and 1212 nm (8251 cm^−1^) as the second overtone of CH stretching for both the corn flour and tapioca stretching. 

### 3.2. Calibration Models Development Base on FT-NIR

#### 3.2.1. Adulteration by Corn Flour

The results of the prediction of level adulteration corn flour in coconut milk utilization benchtop FT-NIR on training and testing data sets are presented in Table 2. The coefficient of determination (*R*^2^) for all architecture network regressors was decreased from training to testing, which was inversely proportional to Bias and RMSE performance, which have increased from training to testing. As can be seen, all types of architecture networks have excellent performance model capability (RPD > 8.1) that can be expected for predictions of future samples. As for comparing the four architecture networks from the deep-learning regressor, the GoogleNET regressor (best RPD = 20.866) possesses much higher detection accuracy than the other regressor. In other words, this study may organize the performance of regressor analysis order according to its RPD as ResNET < Simple CNN < S-AlexNET < GoogleNET. However, the GoogleNET regressor requires a higher epoch than the others.

**Figure 4:**
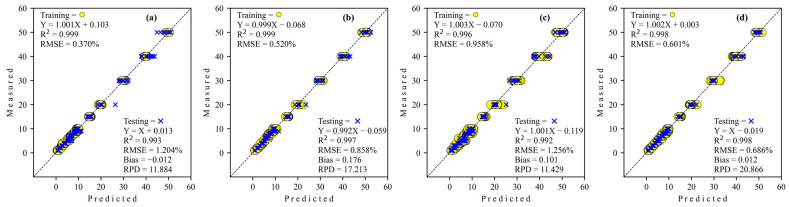
Regression plots obtained by deep learning to detect adulteration of coconut milk by corn flour using FT-NIR. (**a**) Simple CNN; (**b**) S-AlexNET; (**c**) ResNET; and (**d**) GoogleNET.

The regression scatter plots of all architecture networks of deep-learning regression to predict level adulteration corn flour in coconut milk utilization FT-NIR are illustrated in Figure 4. The regression coefficient (slope) for all architecture network regressors decreased from training to testing. The intercept coefficient for simple CNN and GoogleNET is positive, while S-AlexNET and ResNET are negative for training. However, all regressors’ intercept coefficients are negative, except simple CNN in testing.

**Figure 5:**
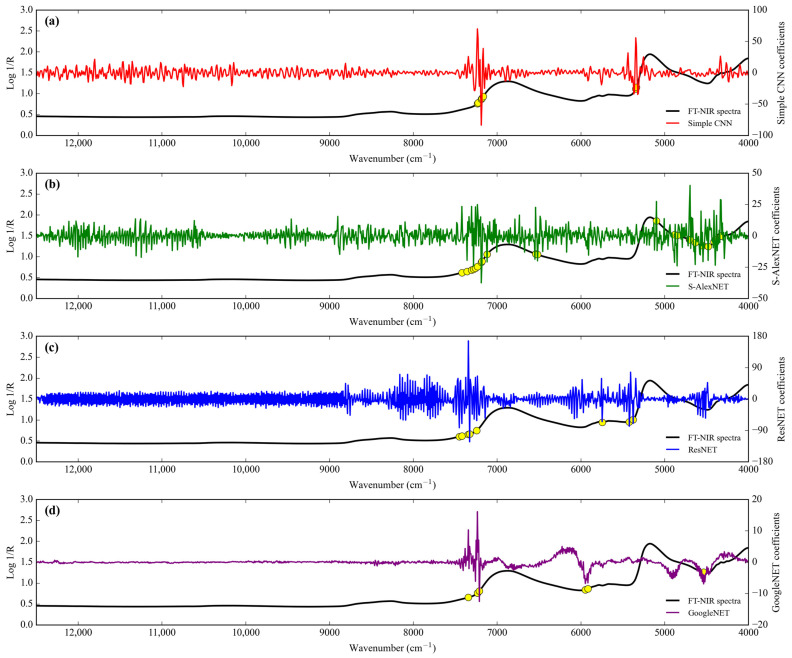
Comparison of the regression coefficients of the four deep-learning calibration approaches of adulteration coconut milk by corn flour using FT-NIR. (**a**) Simple CNN; (**b**) S-AlexNET; (**c**) ResNET; and (**d**) GoogleNET. 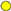 Feature importance.

The range of the simple CNN coefficient from coconut milk adulteration by corn flour using FT-NIR is between 69.996 and −83.475 (Figure 5a). The wavenumbers that have more than score threshold 50% in the range of simple CNN coefficient of architecture are 7236 cm^−1^ (1382 nm), 7228 cm^−1^ (1384 nm), 7190 cm^−1^ (1391 nm), 7182 cm^−1^ (1392 nm), 7167 cm^−1^ (1395 nm), 5346 cm^−1^ (1871 nm), and 5338 cm^−1^ (1873 nm). They have as many as seven wavenumbers of feature importance.

Next, the range of the S-AlexNET coefficient is between 40.231 and −37.471 (Figure 5b). The wavenumbers that have more than score threshold 50% in the range of S-AlexNET coefficient of architecture are 7421 cm^−1^ (1348 nm), 7360 cm^−1^ (1359 nm), 7306 cm^−1^ (1369 nm), 7282 cm^−1^ (1373 nm), 7259 cm^−1^ (1378 nm), 7236 cm^−1^ (1382 nm), 7190 cm^−1^ (1391 nm), 7182 cm^−1^ (1392 nm), 7120 cm^−1^ (1404 nm), 6542 cm^−1^ (1529 nm), 6519 cm^−1^ (1534 nm), 5099 cm^−1^ (1961 nm), 4883 cm^−1^ (2048 nm), 4852 cm^−1^ (2061 nm), 4706 cm^−1^ (2125 nm), 4698 cm^−1^ (2129 nm), 4636 cm^−1^ (2157 nm), 4544 cm^−1^ (2201 nm), 4482 cm^−1^ (2231 nm), 4413 cm^−1^ (2266 nm), 4397 cm^−1^ (2274 nm), 4336 cm^−1^ (2306 nm), and 4328 cm^−1^ (2311 nm). They contain a total of 23 wavenumbers that are of important features.

Also, the range of the ResNET coefficient is between 166.725 and −122.456 (Figure 5c). The wavenumbers that have more than score threshold 50% in the range of ResNET coefficient of architecture are 7452 cm^−1^ (1342 nm), 7421 cm^−1^ (1348 nm), 7344 cm^−1^ (1362 nm), 7329 cm^−1^ (1364 nm), 7244 cm^−1^ (1380 nm), 5747 cm^−1^ (1740 nm), 5423 cm^−1^ (1844 nm), and 5377 cm^−1^ (1860 nm). They consist of a total of eight spectral important features.

Finally, the range of the GoogleNET coefficient is between 16.124 and −12.524 (Figure 5d). The wavenumbers that have more than score threshold 50% in the range of GoogleNET coefficient of architecture are 7344 cm^−1^ (1362 nm), 7236 cm^−1^ (1382 nm), 7213 cm^−1^ (1386 nm), 5948 cm^−1^ (1681 nm), 5917 cm^−1^ (1690 nm), and 4536 cm^−1^ (2205 nm). There are a total of six wavenumbers of important features.

#### 3.2.2. Adulteration by Tapioca Starch

The results of the prediction of level adulteration tapioca starch in coconut milk utilization benchtop FT-NIR on training and testing data sets are presented in Table 3. It is clear that the GoogleNET regressor requires a more increased epoch than the others. The coefficient of determination (*R*^2^) for all architecture network regressors was decreased from training to testing, which is conversely proportional to Bias and RMSE performances, which have increased from training to testing. If focused on RPD, all types of architecture networks have excellent performance model capability (RPD > 8.1), except ResNET (RPD < 3), which shows poor and unreliable performance. As for comparing the four architecture networks from the deep-learning regressor, the GoogleNET regressor (best RPD = 21.421) possesses a much higher prediction than the other regressor. If organized in the best possible way (based on RPD), the performance of deep-learning architecture network regressors of this study can be arranged into GoogleNET > S-AlexNET > Simple CNN > ResNET.

**Figure 6:**
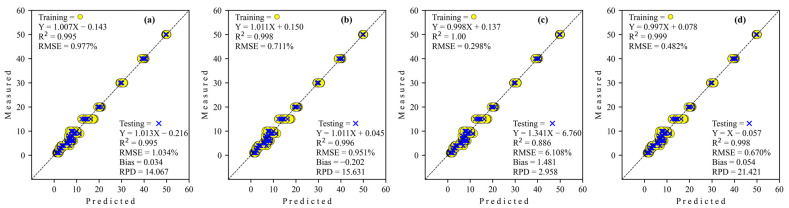
Regression plots obtained by deep learning to detect adulteration of coconut milk by tapioca starch using FT-NIR. (**a**) Simple CNN; (**b**) S-AlexNET; (**c**) ResNET; and (**d**) GoogleNET.

The regression scatter plots of all architecture networks of deep-learning regression to predict level adulteration tapioca starch in coconut milk utilization FT-NIR are shown in Figure 6. The regression coefficient (slope) for all architecture network regressors decreases from training to testing, except S-AlexNET, which decreases very little (more than three decimal places), so the effect is minimal. Also, the intercept coefficient for all regressors is positive, while Simple CNN is negative for training. Inversely proportional to testing, all regressors are negative except S-AlexNET.

**Figure 7:**
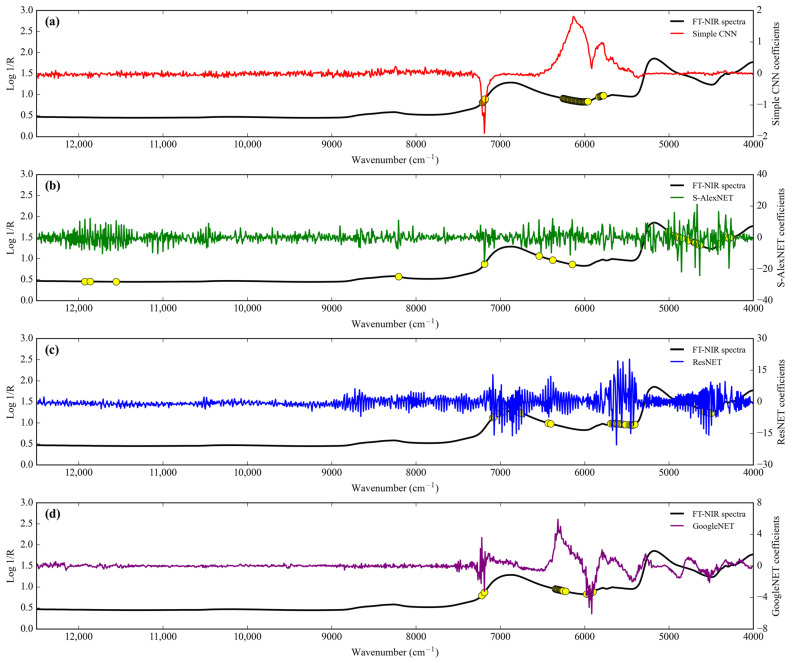
Comparison of the regression coefficients of the four deep-learning calibration approaches to adulteration of coconut milk by tapioca starch using FT-NIR. (**a**) Simple CNN; (**b**) S-AlexNET; (**c**) ResNET; and (**d**) GoogleNET. 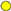 Feature importance.

The simple CNN coefficient range for detecting tapioca starch in coconut milk using FT-NIR is 1.8127 to −1.892. Wavenumbers with more than score threshold 50% of the simple CNN coefficient of architecture as many as 51 important features include 7213–7182 cm^−1^ (1386–1392 nm), 6256–5963 cm^−1^ (1598–1677 nm), and 5832–5778 cm^−1^ (1715–1731 nm) (Figure 7a).

Next, the S-AlexNET coefficient range is from 21.049 to −24.092 (Figure 7b). Wavenumbers with more than score threshold 50% of the S-AlexNET coefficient of architecture include 11,926 cm^−1^ (839 nm), 11,865 cm^−1^ (843 nm), 11,556 cm^−1^ (865 nm), 8208 cm^−1^ (1218 nm), 7190 cm^−1^ (1391 nm), 6542 cm^−1^ (1529 nm), 6380 cm^−1^ (1567 nm), 6148 cm^−1^ (1627 nm), 5007–4690 cm^−1^ (1997–2132 nm), 4667 cm^−1^ (2143 nm), 4636 cm^−1^ (2157 nm), 4413 cm^−1^ (2266 nm), 4328 cm^−1^ (2311 nm), 4289 cm^−1^ (2332 nm), and 4266 cm^−1^ (2344 nm) (a total of 51 important wavenumbers).

Also, the range of the ResNET coefficient is from 20.277 to −20.429. Wavenumbers with more than score threshold of 50% of the ResNET coefficient of architecture as many as 32 importance spectral include 7090 cm^−1^ (1410 nm), 7074 cm^−1^ (1414 nm), 7012 cm^−1^ (1426 nm), 6982–6750 cm^−1^ (1432–1481 nm), 6434–6403 cm^−1^ (1554–1562 nm), 5693–5408 cm^−1^ (1757–1849 nm), and 4621–4498 cm^−1^ (2164–2223 nm) (Figure 7c).

Finally, the GoogleNET coefficient range is from 5.915 to −6.067 (Figure 7d). Wavenumbers with more than a score threshold of 50% of the GoogleNET coefficient of architecture include 7221–7190 cm^−1^ (1385–1391 nm), 6349–6226 cm^−1^ (1575–1606 nm), and 5979–5902 cm^−1^ (1673–1694 nm) (a total of 22 importance wavenumbers).

### 3.3. Calibration Models Development Base on Micro-NIR

#### 3.3.1. Adulteration by Corn Flour

The performances of the different network architectures to predict the level of adulteration of coconut milk by corn flour using Micro-NIR during training and testing are summarized in Table 4. The GoogleNET regressor needs a more significant number of epochs compared to the other regressors. The ResNET regressor is the one that provided the best results (same *R*^2^ with GoogleNET regressor but with the lowest RMSE) during training. The GoogleNET regressor possesses a much higher coefficient of determination (*R*^2^) and lowest RMSE than the other regressor in the test set. This is confirmed by the RPD parameter for the GoogleNET regressor, which has excellent performance model capability (RPD = 31.094). In simpler terms, this study may also arrange the performance of regressor order according to its RPD as Simple CNN < ResNET < S-AlexNET < GoogleNET.

**Figure 8:**
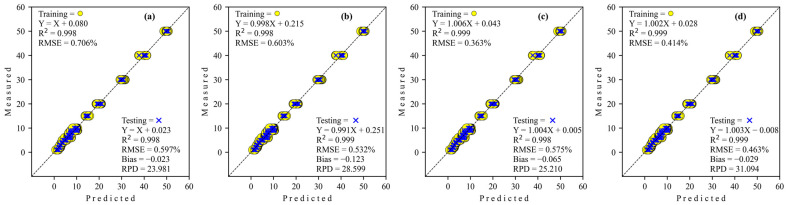
Regression plots obtained by deep learning to detect adulteration of coconut milk by corn flour using Micro-NIR. (**a**) Simple CNN; (**b**) S-AlexNET; (**c**) ResNET; and (**d**) GoogleNET.

All predictive performances from deep-learning regressors in scatter plots to predict the level adulteration of corn flour in coconut milk operating Micro-NIR are shown in Figure 8. The regression coefficient (slope) for S-AlexNET and ResNET regressors decreased from training to testing. Meanwhile, Simple CNN tends to be stable, and GoogleNET overlooks the increase. The intercept coefficient for all regressors, both in training and testing, is positive except for the GoogleNET regressor in the testing stages.

**Figure 9:**
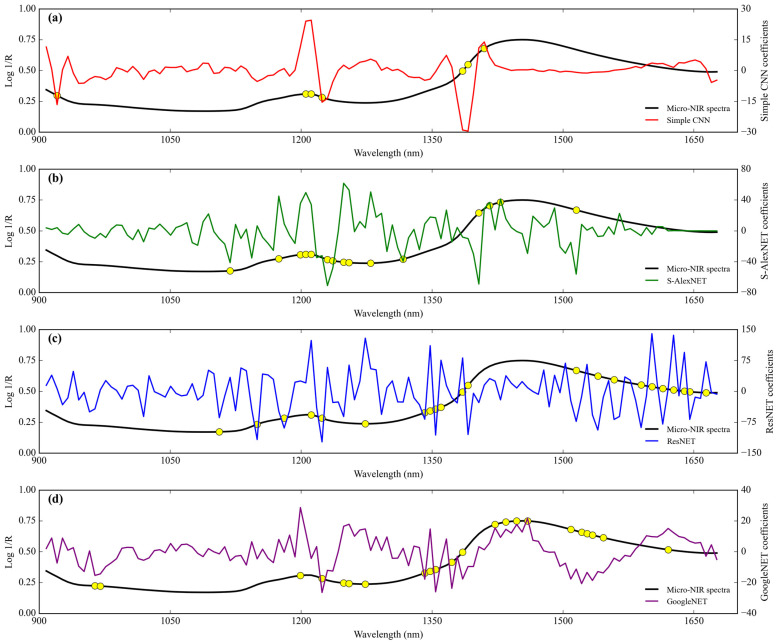
Comparison of the regression coefficients of the four deep-learning calibration approaches of coconut milk adulteration by corn flour using Micro-NIR. (**a**) Simple CNN; (**b**) S-AlexNET; (**c**) ResNET; and (**d**) GoogleNET. 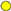 Feature importance.

The range of the simple CNN coefficient from coconut milk adulteration by corn flour using Micro-NIR is between 24.486 and −29.476 (Figure 9a). The wavelengths that have more than score threshold 50% in the range of simple CNN coefficient of architecture (seven wavelengths) are 921 nm (10858 cm^−1^), 1206–1212 nm (8292–8251 cm^−1^), 1224 nm (8170 cm^−1^), 1385 nm (7220 cm^−1^), 1391 nm (7189 cm^−1^), and 1410 nm (7092 cm^−1^). 

Next, the range of the S-AlexNET coefficient is between 61.807 and −70.932 (Figure 9b). The wavelengths that have more than score threshold 50% in the range of S-AlexNET coefficient of architecture (15 wavelengths) are 1119 nm (8937 cm^−1^), 1175 nm (8511 cm^−1^), 1199 nm (8340 cm^−1^), 1205–1212 nm (8299–8251 cm^−1^), 1230–1236 nm (8130–8091 cm^−1^), 1249–1255 nm (8006–7968 cm^−1^), 1280 nm (7813 cm^−1^), 1317 nm (7593 cm^−1^), 1404 nm (7123 cm^−1^), 1416 nm (7062 cm^−1^), 1428 nm (7003 cm^−1^), and 1515 nm (6601 cm^−1^). 

Likewise, the range of the ResNET coefficient is between 140.050 and −122.771 (Figure 9c). The wavelengths that have more than score threshold 50% in the range of ResNET coefficient of architecture are 1106 nm (9042 cm^−1^), 1150 nm (8696 cm^−1^), 1181 nm (8467 cm^−1^), 1212 nm (8251 cm^−1^), 1224 nm (8170 cm^−1^), 1274 nm (7849 cm^−1^), 1342–1360 nm (7452–7353 cm^−1^), 1385–1391 nm (7220–7189 cm^−1^), 1515 nm (6601 cm^−1^), 1540 nm (6494 cm^−1^), 1559 nm (6414 cm^−1^), 1590 nm (6289 cm^−1^), 1602 nm (6242 cm^−1^), 1614 nm (6196 cm^−1^), 1627 nm (6146 cm^−1^), 1639 nm (6101 cm^−1^), 1645 nm (6079 cm^−1^), and 1664 nm (6010 cm^−1^). They contain a total of 22 wavelengths that are of important features. 

Lastly, the range of the GoogleNET coefficient is between 28.666 and −26.421 (Figure 9d). The wavelengths that have more than score threshold 50% in the range of GoogleNET coefficient of architecture are 964–970 nm (10,373–10,309 cm^−1^), 1199 nm (8340 cm^−1^), 1224 nm (8170 cm^−1^), 1249–1255 nm (8006–7968 cm^−1^), 1274 nm (7849 cm^−1^), 1342–1348 nm (7452–7418 cm^−1^), 1354 nm (7386 cm^−1^), 1373 nm (7283 cm^−1^), 1385 nm (7220 cm^−1^), 1422 nm (7032 cm^−1^), 1435 nm (6969 cm^−1^), 1447 nm (6911 cm^−1^), 1459 nm (6854 cm^−1^), 1509 nm (6627 cm^−1^), 1521–1528 nm (6575–6545 cm^−1^), 1534 nm (6519 cm^−1^), 1546 nm (6468 cm^−1^), and 1621 nm (6169 cm^−1^). They have as many as 22 wavelengths that feature importance.

#### 3.3.2. Adulteration by Tapioca Starch

Results of the prediction for the adulteration degree of corn flour in coconut milk using Micro-NIR, as seen in the training and testing data sets, are displayed in Table 5. The coefficient of determination (*R*^2^) for all architecture network regressors is a teeny change from training to testing. The RMSE performance decreased for simple CNN and S-AlexNET while increasing for ResNET and GoogleNET from training to testing. However, Bias performance for simple CNN and ResNET decreased during training to testing, and S-AlexNET and GoogleNET increased during training to testing. Regarding RPD, all architecture networks demonstrate excellent performance model capability (RPD > 8.1), with the best coming from ResNET (RPD = 39.349). To elaborate, this study may organize the best performance of regressor analysis order according to its RPD as ResNET > S-AlexNET > GoogleNET > Simple CNN with GoogleNET regressor requires a higher epoch than others.

**Figure 10:**
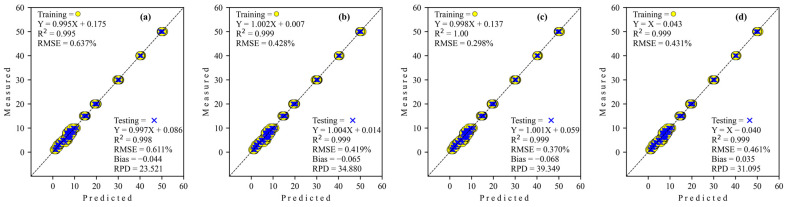
Regression plots obtained to detect adulteration of coconut milk by tapioca starch using Micro-NIR. (**a**) Simple CNN; (**b**) S-AlexNET; (**c**) ResNET; and (**d**) GoogleNET.

The regression scatter plots for predicting the adulteration level of tapioca starch in coconut milk using Micro-NIR with various deep-learning architecture networks are depicted in Figure 10. The regression coefficient (slope) for all architecture network regressors increases during training to testing except GoogleNET, which decreases very little (more than three decimal places), so the effect is minimal. Also, all regressors’ intercept coefficients are positive during training and testing, while GoogleNET is negative for both training and testing.

**Figure 11:**
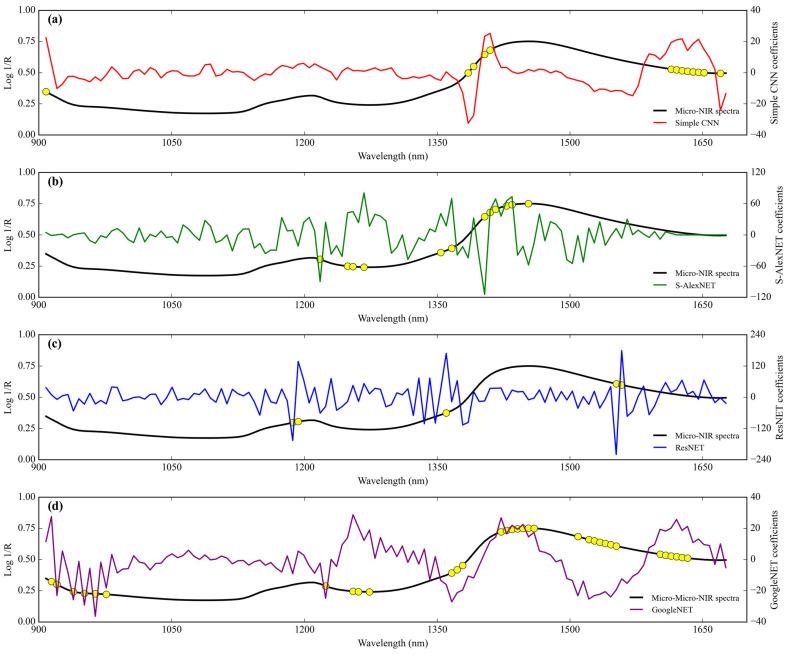
Comparison of the regression coefficients of the four deep-learning calibration approaches of adulteration coconut milk by tapioca starch using Micro-NIR. (**a**) Simple CNN; (**b**) S-AlexNET; (**c**) ResNET; and (**d**) GoogleNET. 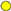 Feature importance.

The simple CNN coefficient range for detecting tapioca starch in coconut milk using Micro-NIR is from 25.180 to −32.352. Wavelengths with more than score threshold 50% of the simple CNN coefficient of architecture with as many as 13 importance features include 908 nm (11013 cm^−1^), 1385 nm (7220 cm^−1^), 1391 nm (7189 cm^−1^), 1404 nm (7123 cm^−1^), 1410 nm (7092 cm^−1^), 1614–1651 nm (6196–6057 cm^−1^), and 1670 nm (5988 cm^−1^) (Figure 11a).

Next, the S-AlexNET coefficient range is from 80.792 to −114.579 (Figure 11b). Wavelengths with more than score threshold 50% of the S-AlexNET coefficient of architecture include 1218 nm (8210 cm^−1^), 1249 nm (8006 cm^−1^), 1255 nm (7968 cm^−1^), 1267 nm (7837 cm^−1^), 1354 nm (7386 cm^−1^), 1367 nm (7315 cm^−1^), 1404 nm (7123 cm^−1^), 1410 nm (7092 cm^−1^), 1416 nm (7062 cm^−1^), 1428 nm (7003 cm^−1^), 1435 nm (6969 cm^−1^), and 1453 nm (6882 cm^−1^) (a total of 12 importance wavelengths).

Likewise, the range of the ResNET coefficient is from 178.959 to −219.921. Wavelengths with more than a score threshold of 50% of the ResNET coefficient of architecture as many as five important spectra include 1187 nm (8425 cm^−1^), 1193 nm (8382 cm^−1^), 1360 nm (7353 cm^−1^), 1552 nm (6443 cm^−1^), and 1559 nm (6414 cm^−1^) (Figure 11c).

Lastly, the GoogleNET coefficient range is from 28.693 to −36.384 (Figure 11d). Wavelengths with more than score threshold of 50% of the GoogleNET coefficient of architecture include 914 nm (10,941 cm^−1^), 921 nm (10,858 cm^−1^), 939 nm (10,650 cm^−1^), 952 nm (10,504 cm^−1^), 964 nm (10,373 cm^−1^), 976 nm (10,246 cm^−1^), 1224 nm (8170 cm^−1^), 1255 nm (7968 cm^−1^), 1261 nm (7930 cm^−1^), 1274 nm (7849 cm^−1^), 1367 nm (7315 cm^−1^), 1373 nm (7283 cm^−1^), 1379 nm (7252 cm^−1^), 1422–1459 nm (7032–6854 cm^−1^), 1509 nm (6627 cm^−1^), 1521–1552 nm (6575–6443 cm^−1^), and 1602–1633 nm (6242–6124 cm^−1^) (a total of 33 importance wavelengths).

## 4. Discussion

This study explored the feasibility of using deep learning to create a rapid, accurate, and robust prediction model to predict adulteration levels of coconut milk by corn flour and tapioca starch using FT-NIR and Micro-NIR spectroscopy. Presently, the non-destructive testing of adulteration in agriculture and food products by NIR spectrometer based on laboratory conditions has been widely introduced. However, the procedures and development of the calibration model under which it can be applied are still limited. The use of deep learning can be a good solution to such problems. Additionally, compared with the results of this study’s use of deep learning, for example, coffee adulteration prediction [4], adulteration in infant formula [12], cow milk fat content adulteration by water [13], and minced beef adulteration [14], we also obtained equally superior prediction results. Furthermore, the operation of the NIR spectrophotometer is simpler and easier to promote.

This article presents a novel deep-learning regression method for quantitative NIR spectrum analysis. This method utilizes four network architectures: Simple convolutional neural network (Simple CNN); S-AlexNET; ResNET; and GoogleNET. However, as is known, a robust NIR model for adulteration detection is hard to achieve due to multiple variation factors, such as different brands and batches of product, the simultaneous existence of several adulterants, temperature, humidity, and spectral drift of light sources, making it hard to obtain stable applications in practice. Therefore, more advanced modeling investigations should be carried out and prepared to evaluate and improve the robustness of the proposed method for the future. However, the limitations of the proposed method should also be further considered and improved. For example, the deep-learning method is much more time-consuming in training than the traditional method and the regular machine-learning algorithm. Therefore, some adulteration studies in food and agriculture products are based on NIR spectroscopy run deep-learning algorithms on graphics processing units (GPU), such as the assurance of tea quality by Yang et al. [17] and detection of adulteration of minced beef by Weng et al. [14]. However, thanks to the fast development of deep-learning hardware, for instance, graphics processing unit (GPU), associative processing unit (APU), tensor processing unit (TPU), and quantum processing unit (QPU), the testing time for the proposed network is acceptable.

As can be observed from Figure 2, the spectral profiles of the degree of coconut milk adulterants by corn flour and tapioca starch were similar and characterized by few substantial differences in peak positions and curve trends. In general, for all the sample adulterants, a few characteristic overlapping peaks contributed by the presence of the main content of coconut milk and adulterant material, including fat, protein, moisture, ash, and carbohydrates. Samples with more adulterant material caused the peak absorbance level to decrease, both for adulteration by corn flour and tapioca starch. This corresponds to the difference in moisture content between coconut milk and adulterant, which causes the free moisture content in the coconut milk to be absorbed by the admixture agent to reach an equilibrium point. As a result, coconut milk that has been adulterated more with a solid adulterant material has a lower absorption spectral ability. This is in line with the report by Malvandi et al. [23], who stated in their study that peak values and their corresponding wavelengths in the NIR region changed as the moisture content altered. Büning-Pfaue [24] emphasized that the strength and weakness of this absorption band come from the strong effect of hydrogen bonds on organic monomers, ions, and polymers in the sample. The presence of content in adulteration coconut milk samples was observed at the main peaks of the following wavebands, both FT-NIR and Micro-NIR: 1210 nm (8262 cm^−1^) related predominantly to CH bond stretching with the second overtone; 1453 nm (6881 cm^−1^) to the first overtone of OH stretching bonds attributed to starch and water; 1728 nm (5786 cm^−1^) and 1764 nm (5670 cm^−1^) to the resonance bands of CH bond stretching with the first overtone; and 1929 nm (5184 cm^−1^) to the CH bonds stretching with the second overtone [25,26,27].

The performance criteria for the prediction model using deep learning in this study were evaluated based on predicting grain chemical composition content. A study by Chu et al. [22] examined the regression model’s capacity to classify RPD in the following manner: less than 3 as a poor or unreliable model, 3.1–4.9 as a fair model, 5.0–6.4 as a good model, 6.5–8.0 as a very good model and more than 8.1 as an excellent model. When comparing the RPD results for the prediction degree of adulteration of coconut milk with corn flour using all the network architectures, it was observed that Micro-NIR was superior to using FT-NIR (for all network architectures). At the same time, all the network architectures were considered excellent models. For tapioca starch in coconut milk case, Micro-NIR performed better than FT-NIR based on RPD among the spectrophotometer to predict the degree of adulteration (Table 6). Subsequently, only ResNET has lower RPD and weak performance (FT-NIR data set) among the network architectures. Regarding the comparison between FT-NIR and Micro-NIR, the models developed for the prediction of coconut milk by adulterant material corn flour and tapioca starch seem to give comparable results using the FT-NIR. The performance of FT-NIR slightly reduced RPD, perhaps due to some factors, including a lack of explanatory variables and collinearity, but fortunately, the RPD obtained is still higher than eight [28,29].

In chemometrics, a limited number of samples with high-dimensional data of features pose common problems like data overfitting and multicollinearity and do not show the main features that are more dominant in the data. Selection of the most important features can lead to the dominant variables in a high-dimensional dataset. In case studies on NIR spectra, this can be represented in many methods, one being by expressing slope coefficients or regression coefficients. According to a study from Palermo et al. [30], regression coefficients can be used to select appropriate predictors according to the magnitude of their absolute values. Even according to a study by Wold et al. [31], in classical chemometric analysis using partial least squares (PLS), small regression coefficients can be ignored as an unimportant term to find the most prominent features and correlate them with the chemical assignment of some structure and bond vibration in the NIR spectroscopy. Additionally, compared with the results of previous research using this approach in analysis in NIR spectroscopy, for example, extra virgin olive oil adulteration prediction by PLS regressor [32], adulteration in quinoa flour by PLS regressor [33], aged-rice adulteration by competitive adaptive reweighted sampling (CARS) combined with PLS regressor [34], and adulterants of notoginseng powder by CARS-PLS regressor [35]. However, in applying advanced chemometrics using machine learning and deep learning, it is still a challenge to demonstrate coefficients that can represent important features.

The regression coefficients from deep-learning algorithms used in this study can be represented using weight coefficients. Even though it is not strictly identical to the regression coefficients in classical chemometric analysis using PLS, at least the weight coefficients of each deep-learning network architecture can indicate variables for each response that are more important than others. Regression coefficients for the case of deep-learning regressors were first introduced by Cui and Fearn [8] and tested on three NIR datasets, including the wheat flour dataset, wheat flour dataset, and protein content dataset. In their study, they randomly draw a few spectra from the dataset and plot the corresponding regression coefficients. This is understandable because deep learning is a non-linear approach, so each spectrum will have its own regression coefficient value, different from the PLS regression coefficient, which has the same value for all sample spectra. However, in this study, because the aim of showing the coefficients of weight of each deep-learning network architecture is to find the dominant features in high-dimensional data, we use all training data spectra. Next, we average the weight coefficients of all the training data spectra, which are called regression coefficients for each deep-learning network architecture. In this study, we apply a threshold score of 50% of the maximum and minimum peaks in the regression coefficient, as shown in Figure 5, Figure 7, Figure 9 and Figure 11. This approach is similar to the system applied in the variable importance in projection (VIP) approach from PLS regression, which applies a threshold score rule that can be data specific, ranging between 0.83 and 1.21 [33,36].

In the case of scanning corn flour in coconut milk (Figure 5 and Figure 9), we can see regression coefficients related to the presence of the structural groups CH and OH. In general, the regression coefficients in this study are in the range of 1200–1500 nm (8333–6667 cm^−1^), which is related to the main wavelength of corn flour found by Jiang and Lu [37]. In the case of scanning with FT-NIR, we can see regression coefficients that overlap with all deep-learning network architectures, at least across nine NIR bands. This starts from wave 7421 cm^−1^ (1348 nm), which is related to the fourth overtone of CH_2_ [25]. Next, waves at 7306–7329 cm^−1^ (1369–1364 nm) and 7344 cm^−1^ (1362 nm) are a combination of CH stretching and CH deformation from CH_3_ [26]. Waves at 7213–7228 cm^−1^ (1386–1384 nm) and 7236–7244 cm^−1^ (1382–1380 nm) correspond to OH stretching from H_2_O [27]. Furthermore, waves at 7167–7190 cm^−1^ (1395–1391 nm) and 7182 cm^−1^ (1392 nm) are related to a combination of CH stretching and CH deformation from CH_2_ [26]. Lastly, the wave at 4536–4544 cm^−1^ (2205–2201 nm) is related to CH stretching and C=O stretching from CHO [26]. However, the regression coefficients that overlap with all deep-learning network architectures that scan using Micro-NIR are 12 NIR bands. The wave was detected starting from 1205–1206 nm (8299–8292 cm^−1^), the fourth overtone of aromatic CH, to 1212 nm (8251 cm^−1^) and 1224 nm (8170 cm^−1^), the second overtone of CH_2_ and CH [25,26]. In addition, waves in the range of 1249–1274 nm (8006–7849 cm^−1^), 1342–1348 nm (7452–7418 cm^−1^), and 1354–1404 nm (7386–7123 cm^−1^) are the fourth overtone beta-diketone, the fourth overtone CH_2_, and the third overtone aldehydes, respectively [25]. Furthermore, waves at 1391 nm (7189 cm^−1^), 1404–1410 nm (7123–7092 cm^−1^), and 1416–1422 nm (7062–7032 cm^−1^) are the representations of combination CH stretching with CH deformation, the first overtone of OH stretching, and a combination CH stretching with CH deformation and the first overtone OH stretching, respectively [26]. Finally, the waves at 1515 nm (6601 cm^−1^), 1540 nm (6494 cm^−1^), and 1614–1621 nm (6196–6169 cm^−1^) are related to the first overtone of CH, the first overtone of OH (starch), and the first overtone of =CH_2_, respectively [25,26].

When examining the tapioca starch in coconut milk (Figure 7 and Figure 11), we see regression coefficients associated with the existence of the structural groups CH, CC, CNO, and OH. The structural groups detected in this sample were relatively slightly different from the adulteration of coconut milk with corn flour. This is due to the composition of the adulterant material, which is also different. The study by Williams [38] was confirmed by Phetpan and Sirisomboon [39], who stated that the peak in the 1400 nm (7143 cm^−1^) region was associated with the glucose molecules in the tapioca starch constituents. The regression coefficients that cover the overlap for all deep learning network architectures when using FT-NIR spectroscopy are in the five NIR spectral bands. Starting from waves 7213–7221 cm^−1^ (1386–1385 nm), 7182–7190 cm^−1^ (1392–1391 nm), 6380–6403 cm^−1^ (1567–1562 nm), 5963–5979 cm^−1^ (1677–1673 nm), and 4621–4636 cm^−1^ (2164–2157 nm), which correspond to the third overtone carbonyl stretching, CH_2_ combination stretching and deformation, the second overtone CC stretching, the first overtone aromatic CH stretching, and the second overtone CNO, respectively [25,26]. Furthermore, the regression coefficients that cover the overlap for all deep-learning network architectures when using Micro-NIR spectroscopy are in the 11 NIR spectral bands. Starting from waves 1255–1276 nm (7968–7837 cm^−1^), 1360–1367 nm (7353–7315 cm^−1^), 1379–1385 nm (7252–7220 cm^−1^), 1404 nm (7123 cm^−1^), and 1410 nm (7092 cm^−1^), which correspond to the third overtone CC stretching, combination stretching, and deformation from CH_3_, the third overtone carbonyl stretching, the third overtone carbonates, and the first overtone of OH stretching, respectively [25,26]. Next, waves at 1416 nm (7062 cm^−1^), 1428 nm (7003 cm^−1^), 1552–1553 nm (6443–6882 cm^−1^), 1614–1621 nm (6196–6169 cm^−1^), and 1627–1633 nm (6146–6124 cm^−1^) are related to combination stretching and deformation of CH_2_, the first overtone of NH stretching, the third overtone carbonyl stretch, the first overtone of OH stretching, the first overtone of =CH_2_ stretching, and the first overtone of CH stretching, respectively [25,26].

This study analyzed important wavelengths by deep learning and found that not all important wavelengths will be the same for all deep-learning network architectures. In addition, even though the FT-NIR wavelength range covers the wavelength range in Micro-NIR, the important wavelength will not be precisely the same for both. However, in the case of FT-NIR and Micro-NIR instruments, it was still found that some important waves overlapped between them. In the case of corn flour, waves 7421 cm^−1^ (1348 nm) and 7167–7190 cm^−1^ (1395–1391 nm) were found in FT-NIR, and 1342–1348 nm (7452–7418 cm^−1^) and 1391 nm (7189 cm^−1^) were found in Micro-NIR. In the case of tapioca starch, the waves at 7213–7221 cm^−1^ (1386–1385 nm) were found in FT-NIR and 1379–1385 nm (7252–7220 cm^−1^) in Micro-NIR. This may be caused by the nature of each regressor, which in the convolutional layer stage can transform the spectra to fit in the following regression scheme. In other words, the regressor from deep learning has carried out automatic preprocessing, as reported by Cui and Fearn [8]. This causes the final shape of each spectrum before the “flatten” to the “dense fully connected” stage to differ according to the output variable. This difference will eventually result in differences in important wavelengths for each deep-learning network architecture. Even though they are different, several wavelengths from all deep-learning network architectures are still the same.

## 5. Conclusions

Deep learning as a novel approach to predict the level of adulteration of coconut milk was successfully developed and tested based on spectra from benchtop FT-NIR and portable Micro-NIR. Models based on FT-NIR spectroscopy to be able to predict the adulteration level of corn flour in coconut milk (1–50%) can be generated using architecture network regressor from deep learning (Simple CNN, S-AlexNET, ResNET, GoogleNET) in the performance ranges of *R*^2^, RMSE, and Bias at their training from 0.996 to 0.999, from 0.370 to 0.958%, and from −0.027 to 0.120, respectively. Next, *R*^2^, RMSE, Bias, and RPD at the testing stage are from 0.992 to 0.998, 0.686 to 1.256%, from −0.012 to 0.176, and from 11.429 to 20.866, respectively. Even though it is still as good, the performance based on the FT-NIR prediction model is still lower than that of Micro-NIR with the same regressor network architecture from deep learning. Performance ranges *R*^2^, RMSE, and Bias at their training using Micro-NIR are from 0.998 to 0.999, from 0.363 to 0.706%, and from −0.053 to −0.183, respectively. At the testing stage, *R*^2^, RMSE, Bias, and RPD are from 0.998 to 0.999, from 0.463 to 0.597%, from −0.023 to 0.123, and from 23.981 to 31.094, respectively.

Relatively similar to the case of the model to predict tapioca starch adulteration in coconut milk, the performance based on the Micro-NIR dataset is better than using FT-NIR with the same regressor network architecture from deep learning. Performance ranges at their training (*R*^2^, RMSE, Bias) and testing (*R*^2^, RMSE, Bias, RPD) using Micro-NIR are from 0.998 to 1.000, from 0.298 to 0.637%, from −0.029 to −0.111, from 0.998 to 0.999, from 0.370 to 0.611%, from −0.035 to −0.068, and from 23.521 to 39.349, respectively. Meanwhile, performance ranges at their training (*R*^2^, RMSE, Bias) and testing (*R*^2^, RMSE, Bias, RPD) using FT-NIR are from 0.892 to 0.999, from 0.482 to 5.850%, from −0.035 to 1.017, from 0.886 to 0.998, from 0.670 to 6.108%, from −0.202 to 1.481, and from 2.958 to 21.421, respectively. 

In closing, the prediction results demonstrated that the proposed architecture from the deep-learning method yielded superior regression performance for the FT-NIR and Micro-NIR to predict the level of adulterants (corn flour and tapioca starch) in coconut milk. While finding that the optimal deep-learning architecture is complex and computationally expensive, implementation and training are straightforward once found. Furthermore, developing deep-learning architectures and applying them are two different study matters that should not be confused. This study also indicated that deep learning for NIR spectroscopy data is less dependent on preprocessing than the classical chemometrics method and still can achieve excellent performance.

## Figures and Tables

**Table 1:** Summary statistics of data for developing a deep-learning model.

Adulteration Material	Instruments	*m*	Training				Testing			
			Min–Max	Mean	SD	*n*	Min–Max	Mean	SD	*n*
Corn flour	FT-NIR	1102	1–50	14.00	14.329	315	1–50	14.00	14.359	135
	Micro-NIR	125	1–50	14.00	14.329	315	1–50	14.00	14.359	135
Tapioca starch	FT-NIR	1102	1–50	14.00	14.329	315	1–50	14.00	14.359	135
	Micro-NIR	125	1–50	14.00	14.329	315	1–50	14.00	14.359	135

*m*, number of features; *n*, number of samples; SD, standard deviation.

**Table 2:** Regression model performance to predict corn flour in coconut milk utilizing FT-NIR.

Regressor	Epoch	Training			Testing			
		*R* ^2^	RMSE	Bias	*R* ^2^	RMSE	Bias	RPD
Simple CNN	8035	0.999	0.370	−0.120	0.993	1.204	−0.012	11.884
S-AlexNET	3300	0.999	0.520	0.076	0.997	0.858	0.176	17.213
ResNET	5929	0.996	0.958	0.027	0.992	1.256	0.101	11.429
GoogleNET	10202	0.998	0.601	−0.037	0.998	0.686	0.012	20.866

**Table 3:** Regression model performance to predict tapioca starch in coconut milk utilizing FT-NIR.

Regressor	Epoch	Training			Testing			
		*R* ^2^	RMSE	Bias	*R* ^2^	RMSE	Bias	RPD
Simple CNN	5633	0.995	0.977	0.039	0.995	1.034	0.034	14.067
S-AlexNET	3000	0.998	0.711	−0.299	0.996	0.951	−0.202	15.631
ResNET	2603	0.892	5.850	1.017	0.886	6.108	1.481	2.958
GoogleNET	10202	0.999	0.482	−0.035	0.998	0.670	0.054	21.421

**Table 4:** Regression model performance to predict corn flour in coconut milk utilizing Micro-NIR.

Regressor	Epoch	Training			Testing			
		*R* ^2^	RMSE	Bias	*R* ^2^	RMSE	Bias	RPD
Simple CNN	6596	0.998	0.706	−0.084	0.998	0.597	−0.023	23.981
S-AlexNET	3300	0.998	0.603	−0.183	0.999	0.532	−0.123	28.599
ResNET	6091	0.999	0.363	−0.129	0.998	0.575	−0.065	25.210
GoogleNET	10128	0.999	0.414	−0.053	0.999	0.463	−0.029	31.094

**Table 5:** Regression model performance to predict tapioca starch in coconut milk using Micro-NIR.

Regressor	Epoch	Training			Testing			
		*R* ^2^	RMSE	Bias	*R* ^2^	RMSE	Bias	RPD
Simple CNN	8872	0.998	0.637	−0.105	0.998	0.611	−0.044	23.521
S-AlexNET	2700	0.999	0.428	−0.029	0.999	0.419	−0.065	34.880
ResNET	7814	1.000	0.298	−0.111	0.999	0.370	−0.068	39.349
GoogleNET	9840	0.999	0.431	0.041	0.999	0.461	0.035	31.095

**Table 6:** Summary of the performance of FT-NIR and Micro-NIR.

Adulteration Material	Instruments	The Best Regressor	RPD
Corn flour	FT-NIR	GoogleNET	20.866
	Micro-NIR	GoogleNET	31.094
Tapioca starch	FT-NIR	GoogleNET	21.421
	Micro-NIR	ResNET	39.349

## Data Availability

Data are contained within the article.

## References

[1] Tansakul A., Chaisawang P. (2006). Thermophysical properties of coconut milk. J. Food Eng..

[2] Lakshanasomya N., Danudol A., Ningnoi T. (2011). Method performance study for total solids and total fat in coconut milk and products. J. Food Compos. Anal..

[3] Azlin-Hashim S., Siang Q.L., Yusof F., Zainol M.K., Mohd Yusof H. (2019). Chemical composition and potential adulterants in coconut milk sold in Kuala Lumpur. Malays. Appl. Biol..

[4] Nallan Chakravartula S.S., Moscetti R., Bedini G., Nardella M., Massantini R. (2022). Use of convolutional neural network (CNN) combined with FT-NIR spectroscopy to predict food adulteration: A case study on coffee. Food Control.

[5] Acquarelli J., van Laarhoven T., Gerretzen J., Tran T.N., Buydens L.M.C., Marchiori E. (2017). Convolutional neural networks for vibrational spectroscopic data analysis. Anal. Chim. Acta.

[6] Sitorus A., Lapcharoensuk R. (2023). A rapid method to predict type and adulteration of coconut milk by near-infrared spectroscopy combined with machine learning and chemometric tools. Microchem. J..

[7] Al-Awadhi M.A., Deshmukh R.R. Detection of Adulteration in Coconut Milk using Infrared Spectroscopy and Machine Learning. Proceedings of the 2021 International Conference of Modern Trends in Information and Communication Technology Industry (MTICTI).

[8] Cui C., Fearn T. (2018). Modern practical convolutional neural networks for multivariate regression: Applications to NIR calibration. Chemom. Intell. Lab. Syst..

[9] Engel J., Gerretzen J., Szymanska E., Jansen J.J., Downey G., Blanchet L., Buydens L.M.C. (2013). Breaking with trends in pre-processing?. TrAC Trends Anal. Chem..

[10] Bengio Y., Courville A., Vincent P. (2013). Representation Learning: A Review and New Perspectives. IEEE Trans. Pattern Anal. Mach. Intell..

[11] Gron A. (2019). Hands-On Machine Learning with Scikit Learn Keras&Tensorflow. 2019.

[12] Liu Y., Zhou S., Han W., Li C., Liu W., Qiu Z., Chen H. (2021). Detection of Adulteration in Infant Formula Based on Ensemble Convolutional Neural Network and Near-Infrared Spectroscopy. Foods.

[13] Said M., Wahba A., Khalil D. (2022). Semi-supervised deep learning framework for milk analysis using NIR spectrometers. Chemom. Intell. Lab. Syst..

[14] Weng S., Guo B., Tang P., Yin X., Pan F., Zhao J., Huang L., Zhang D. (2020). Rapid detection of adulteration of minced beef using Vis/NIR reflectance spectroscopy with multivariate methods. Spectrochim. Acta Part A Mol. Biomol. Spectrosc..

[15] Manley M. (2014). Near-infrared spectroscopy and hyperspectral imaging: Non-destructive analysis of biological materials. Chem. Soc. Rev..

[16] Passos D., Mishra P. (2023). Deep Tutti Frutti: Exploring CNN architectures for dry matter prediction in fruit from multi-fruit near-infrared spectra. Chemom. Intell. Lab. Syst..

[17] Yang J., Wang J., Lu G., Fei S., Yan T., Zhang C., Lu X., Yu Z., Li W., Tang X. (2021). TeaNet: Deep learning on Near-Infrared Spectroscopy (NIR) data for the assurance of tea quality. Comput. Electron. Agric..

[18] Jin B., Zhang C., Jia L., Tang Q., Gao L., Zhao G., Qi H. (2022). Identification of Rice Seed Varieties Based on Near-Infrared Hyperspectral Imaging Technology Combined with Deep Learning. ACS Omega.

[19] Benmouna B., García-Mateos G., Sabzi S., Fernandez-Beltran R., Parras-Burgos D., Molina-Martínez J.M. (2022). Convolutional Neural Networks for Estimating the Ripening State of Fuji Apples Using Visible and Near-Infrared Spectroscopy. Food Bioprocess Technol..

[20] Gulli A., Pal S. (2017). Deep Learning with Keras.

[21] Abadi M., Barham P., Chen J., Chen Z., Davis A., Dean J., Devin M., Ghemawat S., Irving G., Isard M. TensorFlow: A system for Large-Scale machine learning. Proceedings of the 12th USENIX Symposium on Operating Systems Design and Implementation (OSDI ’16).

[22] Chu X., Huang Y., Yun Y.-H., Bian X. (2022). Chemometric Methods in Analytical Spectroscopy Technology.

[23] Malvandi A., Feng H., Kamruzzaman M. (2022). Application of NIR spectroscopy and multivariate analysis for Non-destructive evaluation of apple moisture content during ultrasonic drying. Spectrochim. Acta Part A Mol. Biomol. Spectrosc..

[24] Büning-Pfaue H. (2003). Analysis of water in food by near infrared spectroscopy. Food Chem..

[25] Workman J., Weyer L. (2007). Practical Guide to Interpretive Near-Infrared Spectroscopy.

[26] Osborne B.G., Fearn T., Hindle P.H. (1993). Practical NIR Spectroscopy with Applications in Food and Beverage Analysis.

[27] Conzen J. (2006). Multivariate Calibration: A Practical Guide for Developing Methods in the Quantitative Analytical Chemistry.

[28] Basri K.N., Laili A.R., Tuhaime N.A., Hussain M.N., Bakar J., Sharif Z., Abdul Khir M.F., Zoolfakar A.S. (2018). FT-NIR, MicroNIR and LED-MicroNIR for detection of adulteration in palm oil via PLS and LDA. Anal. Methods.

[29] Lan Z., Zhang Y., Zhang Y., Liu F., Ji D., Cao H., Wang S., Lu T., Meng J. (2021). Rapid evaluation on pharmacodynamics of Curcumae Rhizoma based on Micro-NIR and benchtop-NIR. J. Pharm. Biomed. Anal..

[30] Palermo G., Piraino P., Zucht H.-D. (2009). Performance of PLS regression coefficients in selecting variables for each response of a multivariate PLS for omics-type data. Adv. Appl. Bioinform. Chem..

[31] Wold S., Sjöström M., Eriksson L. (2001). PLS-regression: A basic tool of chemometrics. Chemom. Intell. Lab. Syst..

[32] Jiang H., Chen Q. (2019). Determination of Adulteration Content in Extra Virgin Olive Oil Using FT-NIR Spectroscopy Combined with the BOSS–PLS Algorithm. Molecules.

[33] Wang Z., Wu Q., Kamruzzaman M. (2022). Portable NIR spectroscopy and PLS based variable selection for adulteration detection in quinoa flour. Food Control.

[34] Li Z., Song J., Ma Y., Yu Y., He X., Guo Y., Dou J., Dong H. (2023). Identification of aged-rice adulteration based on near-infrared spectroscopy combined with partial least squares regression and characteristic wavelength variables. Food Chem. X.

[35] Chen H., Tan C., Lin Z., Li H. (2019). Quantifying several adulterants of notoginseng powder by near-infrared spectroscopy and multivariate calibration. Spectrochim. Acta Part A Mol. Biomol. Spectrosc..

[36] Chong I.-G., Jun C.-H. (2005). Performance of some variable selection methods when multicollinearity is present. Chemom. Intell. Lab. Syst..

[37] Jiang H., Lu J. (2018). Using an optimal CC-PLSR-RBFNN model and NIR spectroscopy for the starch content determination in corn. Spectrochim. Acta Part A Mol. Biomol. Spectrosc..

[38] Williams P. (2009). Influence of water on prediction of composition and quality factors: The aquaphotomics of low moisture agricultural materials. J. Near Infrared Spectrosc..

[39] Phetpan K., Sirisomboon P. (2015). Evaluation of the moisture content of tapioca starch using near-infrared spectroscopy. J. Innov. Opt. Health Sci..

